# Ultrasound versus fluoroscopy-guided ureteroscopy for distal ureteric stones in adults

**DOI:** 10.1080/2090598X.2022.2087021

**Published:** 2022-06-20

**Authors:** Ahmed Reda, Yaser Mahmoud Abdelsalam, Mohamed Loay Shehata, Salah El-Din Shaker, Mohammad Abbas Faragallah

**Affiliations:** Urology Department, Faculty of Medicine, Assiut University, Assiut, Egypt

**Keywords:** Fluoroscopy, radiation, stones, ultrasound, ureteroscopy

## Abstract

**Objective:**

To evaluate the safety and efficacy of ultrasound (US) as alternative to fluoroscopy for guidance of ureteroscopy (URS) during treatment of distal ureteric stones in adults.

**Materials and methods:**

This study enrolled 80 patients older than 18 years presented with a single distal ureteric radio-opaque stone of ≤15 mm in longest diameter. Patients were randomized and allocated into two groups: the fluoroscopy group and the ultrasound group (n = 40 patients in each group). Patients with bilateral ureteric stones, solitary kidney, ureteric congenital anomalies, history of failed ureteroscopy, history of ureteric surgery, patients with uremia and pregnant women were excluded. Patients’ demographics, stone characteristics, operative data, stone-free status, hospital stay and complications were evaluated in both groups.

**Results:**

No statistically significant difference between both groups was found regarding patients’ demographics and stone characteristics. Also there was no statistically significant difference in comparing fluoroscopy group versus ultrasound group regarding operative time (29.48 ± 15.3 versus 31.28 ± 18.24 min; P = 0.83), stone-free rate (97.5% versus 95%; P = 1.0), overall complications (15% versus 12.5%; P = 0.75), or hospital stay (1.17 ± 0.6 versus 1.02 ± 0.16 days; P = 0.12). Four patients (10%) in the ultrasound group required the addition of fluoroscopy beside ultrasound.

**Conclusion:**

Ultrasound is effective in guidance during ureteroscopy for distal ureteric stones. It was comparable to fluoroscopy in terms of stone free rate, operative time, overall complications, and hospital stay. However, fluoroscopy must be available to be used when needed.

## Introduction

Urolithiasis is a worldwide health problem that affects about 12% of males and 6% of females during their lifetime [1]. Distal ureteric stones represent 70% of ureteric stones, which are mostly symptomatic [2]. Ureteroscopy (URS) is considered a standard and efficient treatment for distal third ureteric stones, especially in stones with the low possibility of spontaneous passage or failed medical expulsive therapy [3].

During URS, fluoroscopy plays a key role in guiding the entire maneuver, providing more data to endoscopic imaging, and thus making the procedure more secure. However, in comparison with ultrasound, fluoroscopy has many limitations to its routine and safe use. First, and most important, is the associated radiation exposure with its hazards on the patient and the operators taking in consideration the close proximity of the surgeon during URS while the patient is in lithotomy position [4]. Other limitations include the need for a large space in the theater, the need for lucent tables, the ability to recognize only radio-opaque stones, contrast media requirement, technical difficulties with machine manipulations and maintenance, and a higher cost than that of US [5].

US is an efficient guiding imaging in many urological interventions such as percutaneous access of the kidney and urinary bladder. However, it has many limitations such as a second operator is required to handle the US probe and monitor the procedure, the US transducer must be coupled to the patient’s body with maintenance of field sterilization and finally imaging by US might be limited in obese patients or in those with contracted body habitus [6].

This study aimed to assess the safety and efficacy of using US for guiding URS in comparison to fluoroscopy for the treatment of distal ureteric stones in adults.

## Materials and methods

After institutional review board approval, this study was conducted from April 2018 to April 2020. It was a single-center, open-label, randomized, non inferiority, parallel-treatment clinical trial. Included patients were 18 years old or more presented by a single symptomatic distal third ureteric radio-opaque stone with the longest dimension of ≤15 mm. We excluded all patients with bilateral ureteric stones, those with solitary functioning kidney, ureteric congenital anomalies (e.g. double ureter or ectopic ureter), previously failed URS, prior ureteric stenting, previous ureteric surgery such as ureteroneocystostomy, uremic patients and pregnant women. Counseling for participation was provided before recruitment, and written consent was obtained from eligible participants.

**Figure 1.**
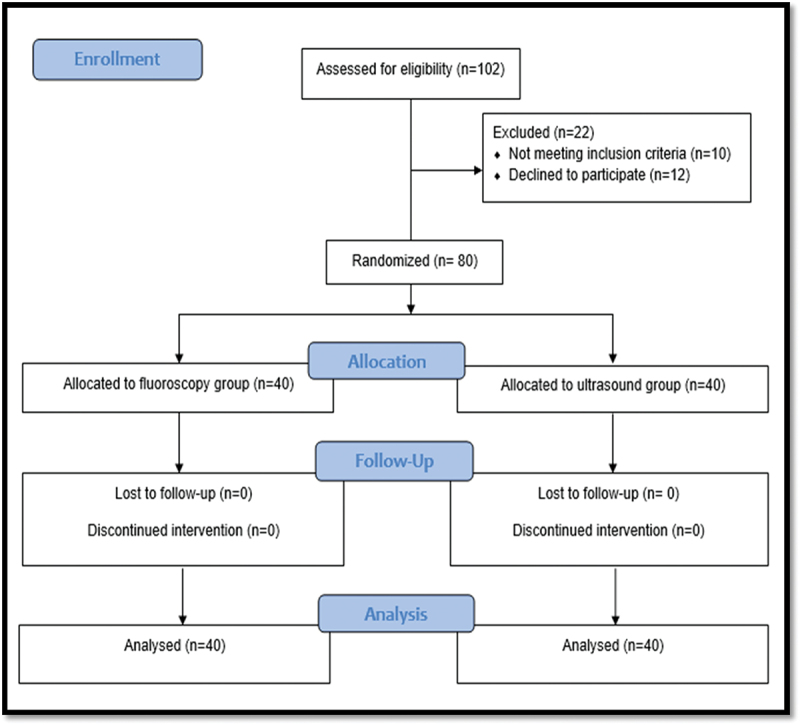
Study flow chart.

Statistical analyses was based on Deter et al study [7]. With a 20% mean difference (delta) of 9 min (45–36), SD of 13 min, a power of 80%, and a significance level of 0.05, the estimated number of patients in each group was 40 (34 + 6) patients assuming a lost-to-follow-up rate of 15%. The sample size calculation was two-tailed and performed using R 4.1.2 (R Core Team 2021, Vienna, Austria). Eligible participants were randomized using a computer-generated table of random numbers with allocation concealment. Allocation concealment was conducted using serially numbered closed opaque envelopes and the study flow chart is illustrated in Figure 1.

All patients eligible for the study underwent complete preoperative evaluation including history, clinical examination, laboratory investigations (needed for surgical fitness), urine analysis with culture and sensitivity, radiological evaluation including pelvic-abdominal US, plain kidneys, ureters and bladder X-ray (KUB), and non-contrast computed tomography (NCCT) of the urinary tract.

The study included two intervention groups, in group I (the control group), patients underwent URS using semi rigid URS (R. Wolf: Diameter 8.5–11.5 Fr, length 31.5 cm with an offset eyepiece), with fluoroscopic guidance (Fluoroscopy C-arm machine, Philips BV Endura, model 718,075, Holland 2018) available throughout the procedure. In group II (the test group), the same steps were applied as for the control group; however, fluoroscopy was replaced with real-time ultrasound (Mindray ultrasound, model DP-5 E, China, 2013, with convex transducer 35C50EA with center frequency of 3.5 MHz).

In both groups, patients received prophylactic antibiotic parenteral third-generation cephalosporin (ceftriaxone 1 gr), if negative culture, or another antibiotic according to culture and sensitivity. The steps for standard URS were carried out for group I, including the following maneuvers under fluoroscopic guidance: insertion of guide-wire, retrograde uretero-pyelography, ureteric dilatation by either Teflon dilators or balloon dilator, stone fragmentation by pneumatic disintegrator, fragments retrieval and stent placement [8]. URS for group II was conducted in a classic and routine manner as in the first group; however, US was used instead for the detection of the following: guide-wire inside the kidney, balloon of the balloon dilator in the distal ureter after being inflated by saline (the deflated balloon could not be detected by US), dilated pelvic-calyceal system, undilated pelvic-calyceal system after being drained, and renal end of the ureteric stent {ureteric catheter or Double-J stent (JJ)}.US was carried out by an assistant urologist who maintained a sterile surgical field during the maneuver. Images were shared and interpreted by the operating urologist as well. Monitoring of the guide-wire, balloon dilator, stone disintegration, and ureteric stent placement relied mainly on endoscopic direct vision. If the guide-wire was not easily introduced at the start of the maneuver, direct access to the ureteric orifice and the intramural part of the ureter was attempted using the 6 Fr ureteroscope (R. Wolf: Diameter 6–7.5 Fr, length 31.5 Cm with an offset eyepiece), if the previous step failed, the operator resorted to fluoroscopy which was also used whenever an obstacle was encountered during the maneuver. Stone free status was defined as absence of measurable residual stone fragments.

For both groups, postoperative management included postoperative analgesics of either ketorolac tromethamine (30 mg intramuscularly or intravenous infusion or paracetamol 1000 mg intravenous infusion given on demand and postoperative antibiotics were ceftriaxone 1 gr or according to preoperative urine culture result. Patients were observed for complications which were classified according to modified Clavien classification system (MCCS) [9]. Patients were discharged 1 day after the operation if no complications occurred.

Postoperative imaging included pelvic–abdominal US and KUB on the day after the procedure to confirm both stone-free status and correct stent position. The ureteric catheter was removed 3 to 5 days after the operation, and the JJ was left for 4–6 weeks postoperatively. The patients were requested to attend a postoperative follow-up visit at the outpatient clinic after 1 week.

## Statistical analysis

Data management and statistical analysis were performed using the Statistical Package for Social Sciences. Data were explored for normality using the Kolmogorov–Smirnov test and Shapiro–Wilk test. Numerical data were summarized using the mean ± standard deviation and ranges. The comparison between the two groups with respect to normally distributed numeric variables was performed using the independent *t*-test. Categorical data were summarized as percentages and frequencies. For categorical variables, differences were analyzed with chi-square test (χ^2^) or Fisher’s exact as appropriate. All *P* values were two-sided. *P* ≤ 0.05 was considered significant.

## Results

There was no statistically significant difference as regard demographic data and preoperative clinical characteristics (Table 1). There was no statistically significant difference as regard operative time, guide-wire used, method of ureteric dilatation (balloon or Teflon dilators), stenting of ureter by JJ or ureteric catheter, and hospital stay (Table 2).

**Table 1.** Patient demographics and baseline characteristics.

Parameter	Group I: Fluoroscopy group (n = 40)	Group II: Ultrasound group (n = 40)	*P-*value
Age in years	43.18 ± 12	43.35 ± 14.83	0.9
Gender Male Female	26 (65) 14 (35)	31 (77.5) 9 (22.5)	0.2
Body mass index, kg/m^2^	25.73 ± 2.74	25.19 ± 4	0.4
History of spontaneous stone passage	4 (10)	0 (0)	0.12^€^
Previous medical expulsive therapy	20 (50)	18 (45)	0.65^Ψ^
History of previous urologic operations	12 (30)	14 (35)	0.63^Ψ^
Stone side Right Left	24 (60) 16 (40)	19 (47.5) 21 (52.5)	0.26*^€^*
Stone size, mm	10.48 ± 2.98	10.25 ± 3.36	0.896*^¥^*
Hydronephrosis No Mild Moderate Marked	6 (15.0) 26 (65.0) 7 (17.5) 1 (2.5)	3 (7.5) 28 (70.0) 6 (15.0) 3 (7.5)	0.54*^Ψ^*

€ = Fisher’s exact test; ¥ = Mann–Whitney *U* test; Ψ = chi-square test.

*P* ≤ 0.05 is considered statistically significant.

**Table 2.** Comparison between the study groups regarding operative time, guide wires used, methods of ureteric dilatation, methods of uretric stenting, and hospital stay periods in days.

Parameter	Group I: Fluoroscopy group (n = 40)	Group II: Ultrasound group (n = 40)	*P* value
Operative time, min*	29.48 ± 15.3	31.28 ± 18.24	0.828
Guide wire** Sensor Zebra Teflon	28 (70%) 5 (12.5%) 7 (17.5%)	31 (77.5%) 4 (10%) 5 (12.5%)	0.74
Method of ureteric dilatation*** Without Teflon Balloon	2 (5%) 33 (88.5%) 5 (12.5%)	3 (7.5%) 32 (80%) 5 (12.5%)	0.74
Method of ureteric stenting*** No stent Ureteric catheter JJ stent	3 (7.5%) 23 (57.5%) 14 (35%)	1 (2.5%) 22 (55%) 17 (42.5%)	0.52
Hospital stay, days*	1.17 ± 0.6	1.02 ± 0.16	0.12

*SD = standard deviation. *P* ≤ 0.05 is considered statistically significant, analysis by Mann–Whitney *U* test.

**P ≤ 0.05 is considered statistically significant, analysis by chi-square test.

***P ≤ 0.05 is considered statistically significant, analysis by Fisher’s exact test

Complications in our study occurred in six (15%) patients in fluoroscopy group and five (12.5%) patients in US group, with no statistically significant difference. These complications included, ureteric mucosal abrasions (two patients in fluoroscopy group and two patients in US group), fever reached to 38°C (one patient in US group) and ureteric bleeding (two patients in fluoroscopy group) all are grade I according to MCCS. The rest of complications were grade III a MCCS and included significant residual stone fragments (one patient in both group) for which second look URS was done, ureteric perforation (one patient in fluoroscopy group) which mandated JJ insertion, and stone retropulsion to the kidney (one patient in US group) which required extracorporeal shock wave lithotripsy (ESWL) later on. There was no statistically significant difference between both groups as regard overall complications (*P* = 0.75).

The stone-free rate was 97.5% in the fluoroscopy group and 95% in the US group, with no statistical significant difference (*P* = 1.0). One patient (2.5%) each in the fluoroscopy and US groups had significant residual ureteric stone fragments that required retreatment in the form of second-look URS (in the first case, the primary maneuver was postponed due to ureteric bleeding which interfered with complete stone retrieval, while in the second case, significant stone fragment was discovered by postoperative imaging). In addition, one patient in the US group required ESWL due to stone retropulsion and upward migration to the kidney.

In the current study, only four patients (10%) in the US group required auxiliary use of fluoroscopy in addition to the US guidance. The reasons for that were failure of identification of the ureteric orifice so the guide-wire was antegradely inserted in one case, failure of free ascent of the guide-wire after being successfully introduced into the ureteric orifice in two cases, so ascending ureterography was done which revealed ureteric kinks and difficulty in JJ insertion at the end of the maneuver in the fourth case. These four cases were not excluded from the statistical analysis of the US group.

## Discussion

URS remains one of the first-line treatment options for ureteric stones. Conventional URS relies on intraoperative fluoroscopy for guidance which exposes both the patient and the operating room staff to harmful ionizing radiation. Hence, there are always attempts to decrease the radiation dose and/or to use an alternative method free of radiation risk especially for children, fetuses, and women of child-bearing age [10]. US is an excellent surrogate for upper urinary tract imaging, as it is radiation free, rapid, and portable. It is a versatile tool and applicable to all age groups and to pregnant women [11].

For the sake of comparison, we selected three previous studies: Deters et al. 2014, Singh et al. 2016, and Mohey et al. 2018; their results are shown in Table 3. From the table, it is obvious that some patient demographics such as age, gender, and BMI were not associated with the process of URS and its results.

**Table 3.** Comparison between the current study and similar studies from the literature.

Authors	Age, years Mean ±SD		Male/Female		BMI(kg/m^2^)		Stone size (mm) Mean ±SD		Operative time (min) Mean ±SD		Complications (%)		URS for residual stone fragments (%)		Hospital stay(days) Mean ±SD	
		US	Fl*	US	Fl*	US	Fl*	US	Fl*	US	Fl*	US	Fl*	US	Fl*	US
Deters et al. (2014)^7^	56	52			29.89	31.12	5.6	5.9	45.7	36.52	16	8	14	14		
Singh et al. (2016)^11^	42.53 ± 14.52	36.12 ± 11.84	21/18	24/17			<10	˂ 10	43.9 ±12.99	45.61 ± 11.62	10.3	7.3	7.7	4.9	2.4 ±0.54	2.5 ± 0.59
Mohey et al. * (2018)^13^	29.5 ± 14.6	28.8 ± 13.3	49/31	41/33	27.6 ± 2.3	28.2 ± 3.3	7.3 ±1.7	7.2 ± 1.5	40.3 ± 6.5	42.4 ± 8.3	8.75	12.2	5	6.8		
Current study	43.18 ± 12	43.35 ± 14.83	26/14	31/9	25.73 ± 2.74	25.19 ± 4	10.48 ±2.98	10.25 ±3.36	29.48 ±15.3	31.28 ±18.24	15	12.5	2.5	2.5	1.17 ± 0.6	1.02 ± 0.16

*The study by Mohey et al. was on fluoroless URS.

In the current study, the size of the stones in both groups was quite similar to a previous study which carried out fluoroless URS with a mean stone size was calculated to be 10.64 ± 3.16 mm (range, 6–17 mm) [12]. Also it is important to know that stone size in the current study was larger than those in previous three studies (<1 cm) [7,11,13] as shown in Table 3 and larger than stone size in a study treated stones in children with an average stone size of 6 mm (range, 4–8 mm) [14].

In our study, there was no significant difference in operative time between the two groups, but the operative time in both groups was lower than that in the previous three studies [7,11,13] as illustrated in Table 3.

In the current study, ureteric dilatation (using either Teflon or balloon dilators) was performed in 95% of the cases in the fluoroscopy group and in 92.5% of the cases in the US group. The inflated balloon by either contrast material or any clear fluid (such as normal saline) could be detected easily by US in the distal ureter, also complete deflation of the balloon could be checked by US. The balloon dilator has two colored radio-opaque landmarks present distal and proximal to the balloon and can be seen by the naked eye, so balloon dilatation of distal ureter can be done under direct vision without the guidance of fluoroscopy and this was consistent with Mandhani et al [15]. However, fluoroscopy detects the contrast material inside the balloon in even few drops. Moreover, it can easily confirm the level of the balloon inside the ureter depending on the two radio-opaque landmarks.

Stenting of the ureter by JJ was carried out in 14 (35%) and 17 (42.5%) patients in the fluoroscopy and ultrasound groups respectively. In comparison to another study, JJ stents were used in only six patients (5.4%) [15] and, this may be attributed to smaller stone size (mean stone size was 8.7 mm versus 10.48 mm in the current study) which required less intensive manipulation by pneumatic disintegration and retrieval. Brisbane and his colleagues concluded that ureteric stent placement without fluoroscopic guidance is feasible, they found that all 25 (100%) ureteric stent placements were performed successfully without the use of fluoroscopy with similar efficacy and safety to that of conventional ureteric stent placement [16] which was consistent with the current study. Also, in a study by Tepeler et al., the ureteric JJ stent was inserted under direct vision in 19 patients (20.4%) [12].

There is no doubt that fluoroscopy is secure method to use during any blind manipulation inside the ureter. Any obstacle, stoppage of ascent, false passage, or coiling of the guide-wire, dilator or stent can be instantaneously and immediately discovered. In the current study only 4 patients (10%) required use of fluoroscopy while it was required for seven patients (7.52%) in Tepeler et al. [12], and for six patients (7.5%) in Mohey et al [13], and the reasons for use in these previous studies were quite similar to our study, rather than other causes such as, impacted stone, stone upward migration to the kidney and duplicated ureters with double collecting systems. Noteworthy, these 4 cases were included in the results of the US group as the use of fluoroscopy was auxiliary (to support the US guidance in a single unexpected obstacle in the entire maneuver) and the stone finally was completely cleared with the guidance of ultrasound. In addition, our analyses were merely based on intention to treat guidance.

Complications in the current study were comparable to a similar study by Tepler et al. [12] as regard both incidence in US group (12.5% and 11.8% respectively) and grade of complications according to MCCS (Grade I,II, III a and Grade I,II respectively), although the method of disintegration in the current study was pneumatic disintegration compared with Holmium laser in Tepler et al. which may justify Grade III a complications in the current study (significant residual stone fragments, stone retropulsion to the kidney which required secondary procedures without general anesthesia). Complication incidence in the current study was higher than that of Singh et al [11] and Mohey et al [13] this may attributed to larger stone size and method of disintegration. In Deters et al. study, although previous stenting of ureter, smaller stone size (< 1 cm), and use of Holmium laser, complications incidence versus that of the current study was comparable in fluoroscopy group (16% versus 15%) but lower in US group (8% versus 12.5%) [7].

In the current study, the stone-free rate in both groups was higher than that of many studies such as, Deters et al., with stone-free rate reached 86% in both groups without significant difference [7],or in Singh et al. stone free rate was 92% in fluoroscopy group and 95% in US group without significant difference [11], or in Mohey et al., the stone-free rate was 93.2% in fluoroscopy group and 95% in US group with no significant difference [13], and Mandhani et al. with stone free rate without the use of fluoroscopy reached 90% [15].

Hospital stay in the current study was mostly less than 2 days. Hospital stays of longer than 2 days were due to postoperative fever and hematuria. Conservative medical treatment was sufficient for management without any need for surgical intervention. The mean duration of hospital stay was longer in the Singh et al. study (2.4 ± 0.54) days for the fluoroscopy group and (2.5 ± 0.59) days for the US group, with no statistically significant difference [11].

A meta-analysis conducted by Subiela et al. concluded that fluoroless URS offers a similar stone-free rate to that provided by fluoroscopy-guided URS without any increase in operative time, hospital stay, secondary procedures, or patient morbidity [2].

Meanwhile, the above data should not be taken to support the assumption that fluoroscopy is unnecessary during URS. However, all URS, if not performed in a fluoroless manner, should be carried out with as low dose of fluoroscopy as possible. Indeed, US-guided URS is safer for patients in general and for patients in specific clinical situations (such as children, during pregnancy, and in females during their child-bearing period). It is more comfortable for the surgeons, anesthesiologists, and all persons in the operative theater, as all can circulate without lead aprons. Unlike fluoroscopy, the following are challenging for US: monitoring guide-wires inside the ureter, Teflon ureteric dilators, ureteric balloon dilators with their radio-opaque marks and level, stone retrieval and fragmentation devices, or minimal extravasation or fluid collection. US-guided URS requires either a radiologist (who is usually not available) or a urologist (who should be well trained for ultrasound use). Finally, URS is an endourologic procedure that essentially depends on direct vision. If the procedure is straightforward and the condition is favorable, URS can be conducted in a fluoroless or even in a sonoless manner.

The main limitation of the current study is that the surgical maneuver was performed by different operators which might affect surgical expertise and the indication of JJ stent. Another limitation was the lack of data about radiation dose and radiation exposure time.

## Conclusions

US guidance of URS is a safe, effective, and radiation free procedure that is comparable with fluoroscopy in terms of guidance without any appreciable difference in stone free rate, operative time, overall complications and hospital stay. However, fluoroscopy should be kept available and on call whenever the procedure is not simple or is complicated.

## Data Availability

The derived data supporting the findings of this study are available from the corresponding author, Mohammad Abbas Faragallah on request.

## Abbreviations

US: Ultrasound

URS: Ureteroscopy

KUB: kidney-ureter-bladder X-ray.

NCCT: non contrast computed tomography

JJ: double J stent

ESWL: extracorporeal shock wave lithotripsy

MCCS: modified Clavien classification system
